# Detection of differentially expressed candidate genes for a fatty liver QTL on mouse chromosome 12

**DOI:** 10.1186/s12863-016-0385-2

**Published:** 2016-06-06

**Authors:** Misato Kobayashi, Miyako Suzuki, Tamio Ohno, Kana Tsuzuki, Chie Taguchi, Soushi Tateishi, Teruo Kawada, Young-il Kim, Atsushi Murai, Fumihiko Horio

**Affiliations:** Department of Applied Molecular Bioscience, Graduate School of Bioagricultural Sciences, Nagoya University, Nagoya, 464-8601 Japan; Division of Experimental Animals, Center for Promotion of Medical Research and Education, Graduate School of Medicine, Nagoya University, Nagoya, 466-8550 Japan; Division of Food Science and Biotechnology, Graduate School of Agriculture, Kyoto University, Uji, 611-0011 Japan; Department of Applied Biosciences, Graduate School of Bioagricultural Sciences, Nagoya University, Furo-cho, Chikusa, Nagoya, 464-8601 Japan

**Keywords:** Genetics, Nutrition, Liver, Lipids, Fatty acids, CD36, Consomic, High-fat diet

## Abstract

**Background:**

The SMXA-5 mouse is an animal model of high-fat diet-induced fatty liver. The major QTL for fatty liver, *Fl1sa* on chromosome 12, was identified in a SM/J × SMXA-5 intercross. The SMXA-5 genome consists of the SM/J and A/J genomes, and the A/J allele of *Fl1sa* is a fatty liver-susceptibility allele. The existence of the responsible genes for fatty liver within *Fl1sa* was confirmed in A/J-12^SM^ consomic mice. The aim of this study was to identify candidate genes for *Fl1sa,* and to investigate whether the identified genes affect the lipid metabolism.

**Results:**

A/J-12^SM^ mice showed a significantly lower liver triglyceride content compared to A/J mice when fed the high-fat diet for 7 weeks. We detected differences in the accumulation of liver lipids in response to the high-fat diet between A/J and A/J-12^SM^ consomic mice. To identify candidate genes for *Fl1sa*, we performed DNA microarray analysis using the livers of A/J-12^SM^ and A/J mice fed the high-fat diet. The mRNA levels of three genes (*Iah1*, *Rrm2*, *Prkd1*) in the chromosomal region of *Fl1sa* were significantly different between the strains. *Iah1* mRNA levels in the liver, kidney, and lung were significantly higher in A/J-12^SM^ mice than in A/J mice. The hepatic *Iah1* mRNA level in A/J-12^SM^ mice was 3.2-fold higher than that in A/J mice. To examine the effect of *Iah1* on hepatic lipid metabolism, we constructed a stable cell line expressing the mouse Iah1 protein in mouse hepatoma Hepa1-6 cells. Overexpression of Iah1 in Hepa1-6 cells suppressed the mRNA levels of *Cd36* and *Dgat2*, which play important roles in triglyceride synthesis and lipid metabolism.

**Conclusions:**

These results demonstrated that *Fl1sa* on the proximal region of chromosome 12 affected fatty liver in mice on a high-fat diet. *Iah1* (isoamyl acetate-hydrolyzing esterase 1 homolog) was identified as one of the candidate genes for *Fl1sa*. This study revealed that the mouse *Iah1* gene regulated the expression of genes related to lipid metabolism in the liver.

**Electronic supplementary material:**

The online version of this article (doi:10.1186/s12863-016-0385-2) contains supplementary material, which is available to authorized users.

## Background

Fatty liver is associated with dyslipidemia, type 2 diabetes mellitus, and obesity. The SMXA-5 mouse is an animal model of high fat diet (HFD)-induced type 2 diabetes and fatty liver [1]. The SMXA-5 strain is an SMXA-recombinant inbred (RI) strain established from breeding between SM/J mice and A/J mice [2], and thus the SMXA-5 genome is a mosaic genome derived from SM/J mice and A/J mice. On a commercial chow diet, SM/J mice and A/J mice did not show hyperglycemia and impaired glucose tolerance (IGT), but SMXA-5 mice showed IGT [3]. On a high-carbohydrate diet, SM/J mice and A/J mice were resistant to IGT and fatty liver compared with SMXA-5 mice [1]. On the HFD, although the traits were deteriorated in all strains, both parental strains were without either diabetes or fatty liver. But SMXA-5 mice developed type 2 diabetes and fatty liver on the HFD. It was speculated that the parental strains possess the latent susceptibility loci for type 2 diabetes and fatty liver [1]. We previously performed a search of genetic factors for fatty liver in SMXA-5 mice by quantitative trait locus (QTL) analysis in (SM/J × SMXA-5)F2 intercross mice [4]. We detected several QTLs for liver weight, liver total lipids, liver total cholesterol (TC), and liver triglycerides (TG) on mouse chromosomes 2, 6, 10, 11, 12, and 17. On chromosome 12, a significant QTL for liver TG and highly significant QTLs for relative liver weight/liver lipid content were detected near D12Mit58 (17.1 Mb) and D12Mit270 (32.3 Mb), respectively. Chromosome 12 of SMXA-5 had the A/J-derived genome from the centromere to 54.1 Mb, and the SM/J-derived genome from 54.1 Mb to the telomere. The major QTL for fatty liver on chromosome 12 was designated *Fl1sa* (*fattyliver1in theSMXARI strains*). The A/J allele of *Fl1sa* (on centromere-54.1 Mb) contributed to an increase in lipid accumulation in the liver. The effect of *Fl1sa* was confirmed in A/J-12^SM^ chromosomal substitution (consomic) mice [4]. On the HFD, A/J-12^SM^ consomic mice that possessed the SM/J allele of *Fl1sa* showed lower lipid accumulation in the liver than A/J mice.

In this study, we first attempted to determine the time period when the differences in liver TG accumulation between A/J mice and A/J-12^SM^ mice appeared. The effect of the responsible gene(s) for *Fl1sa* that existed on the A/J-derived region of chromosome 12 (centromere-54.1 Mb) in SMXA-5 emerged after 7 weeks of feeding with the HFD. Secondly, we tried to identify candidate genes involved in the *Fl1sa-*regulated control of lipid accumulation in the liver by DNA microarray analysis. We identified only three genes that had significantly different expression levels in the liver between A/J-12^SM^ and A/J mice. The function of one of these candidate genes, mammalian *Iah1*, had not been reported, although the yeast IAH1 protein is known to show esterase activity [5, 6]. Thirdly, to clarify the function of mouse Iah1, we investigated lipid metabolism in the cells stably overexpressing the mouse *Iah1* gene.

## Results

### Phenotypic analyses of A/J-12^SM^ consomic mice at 3, 7, or 11 weeks of feeding with the HFD (Fig. 1 and Table 1)

**Fig. 1:**
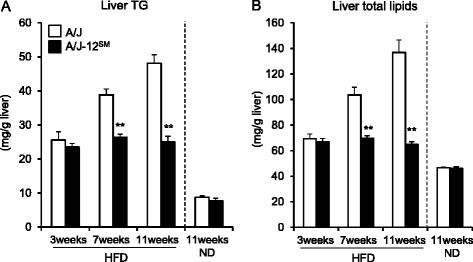
Liver TG and total lipids of A/J mice and A/J-12^SM^ consomic mice at 3, 7, or 11 weeks of feeding with the high-fat diet. **a** Liver TG and (**b**) total lipids of A/J mice and A/J-12^SM^ mice at 3, 7, or 11 weeks of feeding with the HFD. ND, 11 weeks of feeding with the ND (*n* = 8–10; **P < 0.01 versus A/J mice under the same experimental conditions)

**Table 1:** Body weight, body mass index, food intake, and body composition of A/J and A/J-12^SM^ strains fed the high-fat diet for 7 weeks

	A/J (*n* = 9)	A/J-12^SM^ (*n* = 10)	*P*-value
Initial body weight (g)^a^	21.6 ± 0.4	21.3 ± 0.4	NS
Final body weight (g)^b^	36.2 ± 1.0	34.4 ± 0.7	NS
Body mass index (g/cm^2^)	0.311 ± 0.006	0.301 ± 0.006	NS
Food intake (g/week)	20.3 ± 0.3	20.0 ± 0.3	NS
Weights of tissue (g/100 g bw)			
Liver	4.00 ± 0.06	3.55 ± 0.08**	0.0003
Subcutaneous fat^c^	3.30 ± 0.19	3.21 ± 0.15	NS
Epididymal fat	5.21 ± 0.14	5.56 ± 0.24	NS
Retroperitoneal fat	1.45 ± 0.09	1.39 ± 0.09	NS
Mesenteric fat	2.75 ± 0.14	2.47 ± 0.12	NS
Blood glucose (mg/dl)	224.5 ± 5.5	192.7 ± 7.8**	0.0047
Serum insulin (ng/ml)	1.81 ± 0.23	1.08 ± 0.12*	0.0109

Each value is expressed as the mean ± SEM

*P < 0.05, **P < 0.01, significant difference from the value of A/J by Student’s *t*-test

^a^Initial body weight was measured at 6 weeks of age (at 0 weeks of feeding with the HFD)

^b^Final body weight was measured at 13 weeks of age (at 7 weeks of feeding with the HFD)

^c^Subcutaneous fat was defined as fat pads below the root of the forefoot on one side of the body

*NS* not significant

**Fig. 2:**
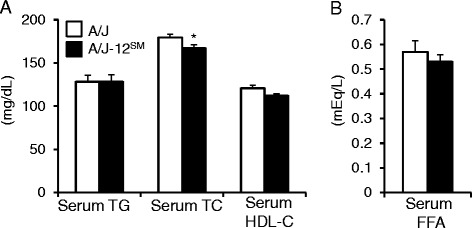
Serum lipids of A/J mice and A/J-12^SM^ consomic mice at 7 weeks of feeding with the high-fat diet. **a** Serum TG, TC, HDL-C, and (**b**) serum FFA levels of A/J and A/J-12^SM^ mice at 7 weeks of feeding with the HFD (*n* = 8–10; *P < 0.05 versus A/J mice)

At 11 weeks of feeding with the normal diet (ND), the body weights (A/J, 29.3 ± 0.6 g; A/J-12^SM^, 27.4 ± 0.9 g), serum TG levels (A/J, 101 ± 8 mg/dl; A/J-12^SM^, 119 ± 7 mg/dl) and serum TC levels (A/J, 100 ± 3 mg/dl; A/J-12^SM^, 106 ± 7 mg/dl) were similar between A/J mice and A/J-12^SM^ mice. Liver TG and liver total lipids in A/J-12^SM^ mice were significantly lower than those in A/J mice at 11 weeks of feeding with the HFD (Fig. 1a and b). These results were consistent with our previous study [4]. However, there were no significant differences in liver TG and liver total lipids between A/J mice and A/J-12^SM^ mice at 11 weeks of feeding with the ND (Fig. 1a and b). The levels of liver total lipids in mice fed the ND were markedly lower than those in mice fed the HFD. These results indicate that *Fl1sa,* the locus for liver lipids accumulation on chromosome 12, affected HFD-induced fatty liver. Therefore, we attempted to determine the time period when the differences of liver TG accumulation between A/J mice and A/J-12^SM^ mice appeared. *Fl1sa* was detected in (SM/J × SMXA-5)F2 intercross mice at 7 weeks of feeding with the HFD [4]. In this study, we analyzed the traits in A/J-12^SM^ mice and A/J mice at 3 or 7 weeks of feeding with the HFD. Feeding with the HFD increased the content of liver TG and liver total lipids compared to feeding with the ND (Fig. 1a and b), but there were no differences between A/J mice and A/J-12^SM^ mice at 3 weeks of feeding with the HFD. The levels of liver TG and liver total lipids in A/J mice increased from 3 to 7 weeks of feeding, but not in A/J-12^SM^ mice. At 7 weeks of feeding, A/J-12^SM^ mice had significantly lower contents of liver TG and of liver total lipids than A/J mice. At 7 weeks of feeding with the HFD, the body weight, body mass index, total food intake, and the weights of each type of adipose tissues (subcutaneous fat, epididymal fat, retroperitoneal fat, and mesenteric fat) did not differ between A/J mice and A/J-12^SM^ mice (Table 1). The liver weight in A/J-12^SM^ mice was significantly lower than that in A/J mice. In addition, the blood glucose and serum insulin concentrations in A/J-12^SM^ mice were lower than those in A/J mice (Table 1). Serum TG, serum HDL-C, and serum free fatty acids (FFA) concentrations in A/J-12^SM^ mice were similar to those in A/J mice (Fig. 2a and b). However, the serum TC level in A/J-12^SM^ mice was lower than that in A/J mice (Fig. 2a).

### Hepatic gene expression analyses between A/J and A/J-12^SM^ consomic mice (Table 2 and Fig. 3a)

**Table 2:** Genes differentially expressed between the livers of A/J mice and A/J-12^SM^ mice on chromosome 12

Probe ID	Gene symbol	Description	Position (bp)	GenBank	RefSeq	*P*-value	Fold change
**1454898_s_at**	**Iah1**	**isoamyl acetate-hydrolyzing esterase 1 homolog (S. cerevisiae)**	**21316392–21323605**	**AU016407**	**NM_026347**	**0.00020**	**4.803**
1459274_at	Gpr135	G protein-coupled receptor 135	72069618**–**72070991	AV221890	NM_181752	0.00029	2.016
**1429119_at**	**Iah1**	**isoamyl acetate-hydrolyzing esterase 1 homolog (S. cerevisiae)**	**21316392–21323605**	**AK005287**	**NM_026347**	**0.00059**	**2.899**
1416951_a_at	Atp6v1d	ATPase, H+ transporting, V1 subunit D	78842989**–**78861638	NM_023721	NM_023721	0.00157	0.492
1443933_at	Tc2n	tandem C2 domains, nuclear	101645443**–**101718523	BB548141	NM_028924	0.00163	0.371
**1437608_x_at**	**Ywhaq**	**tyrosine 3-monooxygenase/tryptophan 5-monooxygenase activation protein, theta polypeptide**	**21390071–21417637**	**BB414446**	**NM_011739**	**0.00183**	**3.096**
1430536_a_at	Erh	enhancer of rudimentary homolog (Drosophila)	80634022**–**80644341	BB071632	NM_007951	0.00218	0.827
1439045_x_at	Tc2n	tandem C2 domains, nuclear	101645443**–**101718523	AV376747	NM_028924	0.00245	0.038
1421139_a_at	Zfp386	zinc finger protein 386 (Kruppel-like)	116047724**–**116063360	NM_019565	NM_001004066 ; NM_019565	0.00267	0.292
**1434437_x_at**	**Rrm2**	**ribonucleotide reductase M2**	**24708241–24714146**	**AV301324**	**NM_009104**	**0.00340**	**0.546**
1446643_at	5330409N07Rik	RIKEN cDNA 5330409 N07 gene	98444816**–**98448333	BB022219	AK030416	0.00422	1.936
1418587_at	Traf3	Tnf receptor-associated factor 3	111166370**–**111267153	U21050	NM_011632	0.00423	0.861
1420491_at	Eif2s1	eukaryotic translation initiation factor 2, subunit 1 alpha	78861819**–**78887010	BC016497	NM_026114	0.00473	4.125
1428810_at	2700097O09Rik	RIKEN cDNA 2700097O09 gene	55045661**–**55080110	AK012621	NM_028314	0.00573	1.337
**1447623_s_at**	**Prkd1**	**protein kinase D1**	**50341231–50649223**	**AV297026**	**NM_008858**	**0.00660**	**0.413**
1449110_at	Rhob	ras homolog gene family, member B	8497763**–**8499985	BC018275	NM_007483	0.00676	1.746
1451146_at	Zfp386	zinc finger protein 386 (Kruppel-like)	116047724**–**116063360	BC004747	NM_001004066 ; NM_019565	0.00692	0.266
**1434700_at**	**G2e3**	**G2/M-phase specific E3 ubiquitin ligase**	**51348061–51376986**	**BM123748**	**NM_001015099**	**0.00809**	**0.636**
1444164_at	Prpf39	PRP39 pre-mRNA processing factor 39 homolog (yeast)	65036333**–**65063386	BG068268	NM_177806	0.00821	0.378
1447341_at	Esyt2	extended synaptotagmin-like protein 2	116281222**–**116373096	BE456208	NM_028731	0.00859	1.141
1454609_x_at	Irf2bpl	interferon regulatory factor 2 binding protein-like	86880703**–**86884814	BB770958	NM_145836	0.00928	1.464
**1420830_x_at**	**Ywhaq**	**tyrosine 3-monooxygenase/tryptophan 5-monooxygenase activation protein, theta polypeptide**	**21390071–21417637**	**NM_011739**	**NM_011739**	**0.00944**	**1.573**
1421430_at	Rad51b	RAD51 homolog B	79297351**–**79508656	NM_009014	NM_009014	0.00982	2.596
1436438_s_at	Dcaf5	DDB1 and CUL4 associated factor 5	80335847**–**80436601	BM234499	NM_177267	0.00984	1.410

Bold genes exist in the chromosomal region of *Fl1sa*. The fold change was calculated by the gene expression level in A/J-12^SM^ relative to that in A/J mice

**Fig. 3:**
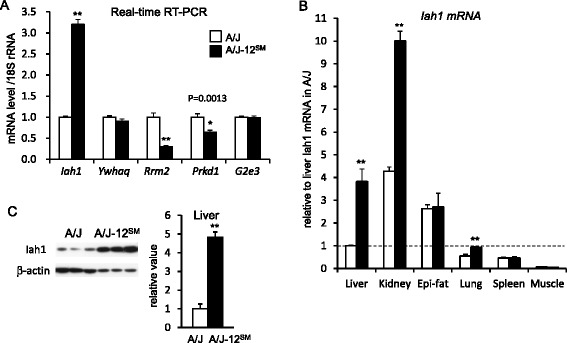
Gene expression levels and protein levels of candidate genes for *Fl1sa*. **a** The hepatic mRNA levels of genes identified from a DNA microarray by real-time RT-PCR analyses (*n* = 9–10; *P < 0.05, **P < 0.01 versus A/J mice). **b** The tissue distribution of *Iah1* mRNA levels in A/J and A/J-12^SM^ mice at 7 weeks of feeding with the HFD (*n* = 4–5; *P < 0.05 versus A/J mice). **c** The hepatic levels of Iah1 protein in A/J and A/J-12^SM^ mice at 7 weeks of feeding with the HFD by Western blotting (*n* = 4; **P < 0.01 versus A/J mice)

Total RNA was extracted from the livers of A/J mice or A/J-12^SM^ consomic mice fed the HFD for 7 weeks. We performed DNA microarray analysis using total RNA from the liver and compared the levels of gene expression between A/J and A/J-12^SM^. On chromosome 12, we detected 20 genes differentially expressed between A/J mice and A/J-12^SM^ mice (*P* < 0.01) (Table 2). Five genes (*Iah1*, *Ywhaq*, *Rrm2*, *Prkd1*, and *G2e3*) exist in the chromosomal region of *Fl1sa* (centromere-54.1 Mb). Using a real-time RT-PCR (qPCR) method, we confirmed that the gene expression levels of *Iah1* (isoamyl acetate-hydrolyzing esterase 1 homolog (*S. cerevisiae*), 21.3 Mb), *Rrm2* (ribonucleotide reductase M2 subunit, 24.7 Mb*)*, and *Prkd1* (protein kinase D1, 50.3 Mb) were significantly different between A/J mice and A/J-12^SM^ mice (Fig. 3a). However, the *Ywhaq* and *G2e3* mRNA levels in A/J-12^SM^ mice did not differ from those in A/J mice. The hepatic *Iah1* mRNA level in A/J-12^SM^ mice was 3.2-fold higher than that in A/J mice. In contrast, the hepatic *Rrm2* mRNA level in A/J-12^SM^ mice (0.30 ± 0.03) was significantly lower than that in A/J mice (1.00 ± 0.10). The *Prkd1* mRNA level in A/J-12^SM^ mice (0.64 ± 0.05) was also significantly lower than that in A/J mice (1.00 ± 0.08).

### Tissue distributions of mouse Iah1 expression (Fig. 3b, c)

Among the three genes that were confirmed to show a change of hepatic mRNA levels, we focused on the *Iah1* gene as a candidate gene for *Fl1sa*. We chose this gene because the *Iah1* mRNA level in the DNA microarray showed the lowest *P*-value and the Iah1 protein was expected to have the esterase activity (Table 2).

We compared the *Iah1* mRNA level among tissues (liver, kidney, epididymal fat, lung, spleen, and muscle) by using the qPCR method (Fig. 3b). The *Iah1* mRNA levels in the liver, kidney, and lung were significantly higher in A/J-12^SM^ mice than in A/J mice. In contrast, the *Iah1* mRNA levels in epididymal fat, spleen, and muscle were similar between A/J mice and A/J-12^SM^ mice. In both A/J mice and A/J-12^SM^ mice, the *Iah1* mRNA level in the kidney was the highest among the tissues. The hepatic Iah1 protein level in A/J-12^SM^ mice was about 4.8-fold higher than that in A/J mice (Fig. 3c).

### Lipid metabolism in Hepa1-6 cells stably overexpressing mouse Iah1 (Fig. 4)

**Fig. 4:**
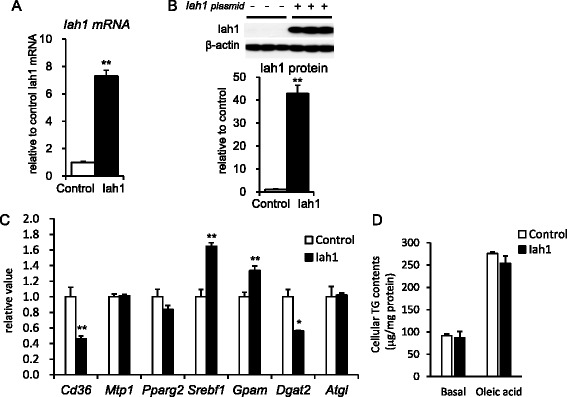
Overexpression of mouse Iah1 in Hepa1-6 cells. **a** The Iah1 mRNA level in non-transfected (control) and mouse *Iah1* cDNA-transfected (Iah1) cells. Iah1 cells were shown to stably overexpress mouse Iah1 protein. **b** The Iah1 protein level in control cells and mouse Iah1 cells. **c** The mRNA levels of genes related to lipid metabolism (*n* = 4–5; *P < 0.05, **P < 0.01 versus A/J mice). **d** The cellular TG contents in non-transfected (control) and mouse *Iah1* cDNA-transfected (Iah1) cells. The treatment medium consisted of serum-free medium plus 0.333 mM oleic acid and was administered to the treatment group for 48 h. The basal group was cultured in serum-free medium without oleic acid. The analyses were performed in duplicate, and at least two independent experiments were performed. The data were analyzed by two-way ANOVA. Oleic acid effect, *P* < 0.05; Iah1 effect, not significant; Oleic acid × Iah1 (interaction) effect, not significant

We transfected mouse *Iah1* cDNA derived from SM/J into Hepa1-6 cells and obtained Hepa1-6 cells stably overexpressing the mouse Iah1 protein (Iah1-cells). The mRNA level and protein level of Iah1 in the non-transfected cells (control cells) could be detected by the qPCR method and western blotting (Fig. 4a and b). The protein level of Iah1 in the Iah1-overexpressed cells was about 42-fold higher than that in the control cells (Fig. 4b). To investigate the effect of stably overexpressing mouse Iah1 protein on lipid metabolism in hepatocytes, we performed an analysis of the mRNA levels of lipid metabolism-related genes (Fig. 4c). The mRNA levels of *Cd36* (fatty acid transporter) in the Iah1-cells were significantly lower than those in the control cells. The mRNA levels of *Mtp1* (microsomal transfer protein 1) did not differ between the control and the Iah1-cells. The genes *Pparg2* (peroxisome proliferator-activated receptor γ), *Srebf1* (sterol regulatory element binding protein 1), *Gpam* (glycerol-3-phosphate acyltransferase, mitochondrial; *Gpat*, synonymous gene symbol), and *Dgat2* (acyl-CoA:diacylglycerol acyltransferase 2) regulate fatty acid and TG synthesis. The mRNA levels of *Srebf1* and *Gpam* were higher in the Iah1-overexpressed cells compared to the control cells. In contrast, the mRNA level of *Dgat2* was significantly lower in the Iah1-overexpressed cells than in the control cells. The mRNA level of *Atgl* (adipose triglyceride lipase), which codes the protein of hepatic major TG lipase, was not changed. In the absence of oleic acid treatment (basal condition), the cellular TG contents did not differ between the control cells and Iah1-cells (Fig. 4d). After treatment with oleic acid, the cellular TG contents in both cell lines were increased. However, overexpression of Iah1did not change the cellular TG contents.

## Discussion

We previously mapped a highly significant QTL for fatty liver (*Fl1sa*) to mouse chromosome 12 in the SMXA-5 mouse, a mouse model for HFD-induced fatty liver and type 2 diabetes. To confirm the effect of this QTL, we constructed A/J-12^SM^ consomic mice. Analysis of the A/J-12^SM^ consomic mice revealed that the SM/J allele on chromosome 12 was resistant to fatty liver [4]. In this study, to elucidate the genetic basis of *Fl1sa,* we first investigated fatty liver-related traits in A/J-12^SM^ consomic mice (Table 1, Figs. 1 and 2). The phenotypic analysis of A/J-12^SM^ mice showed that the SM/J segment of chromosome 12 contributed to the lower values in liver weight, liver lipids content, blood glucose concentration, and serum insulin concentration. We previously mapped the QTLs for glucose tolerance, non-fasting blood glucose level, and serum insulin levels to the proximal region of chromosome 12 [7]. On these QTLs of chromosome 12, the A/J allele increased the values of each phenotype. Therefore, the results for A/J-12^SM^ consomic mice confirmed that chromosome 12 contained not only genes involved in the regulation of liver lipid metabolism, but also several genes regulating glucose metabolism and insulin sensitivity. Significant differences in the liver lipid contents between A/J mice and A/J-12^SM^ consomic mice appeared in the animals fed an HFD for 11 weeks, but not in those on the ND (Fig. 1). These results showed that the A/J-12^SM^ consomic mice were resistant to HFD-induced fatty liver, and demonstrated that mouse chromosome 12 contained some of the genes responsible for HFD-induced fatty liver. Next, in order to identify these genes, we compared the hepatic gene expression profile between A/J mice and A/J-12^SM^ consomic mice fed the HFD, by using a DNA microarray analysis. We identified three candidate genes (*Iah1*, *Rrm2*, *Prkd1*) which had significantly different levels of expression between A/J mice and A/J-12^SM^ consomic mice within the chromosomal region of *Fl1sa* (centromere-54.1 Mb). RRM2 is one of the subunits of the ribonucleotide reductase holoenzyme. The ribonucleotide reductase, which is a rate-limiting enzyme in the production of 2’-deoxyribonucleoside 5’-triophosphates (dNTPs), is required for DNA synthesis. In a human study, expression of the *Rrm2* gene was shown to be increased in various types of cancer [8]. Protein kinase D1 (PKD1), which is coded by the *Prkd1* gene, belongs to the family of stress-activated serine/threonine kinases. PKD1 is activated by the signal via Gq-coupled receptors and protein kinase C, and then phosphorylates various proteins involved in cell growth, apoptosis, adhesion, and angiogenesis [9]. Dysregulation of PKD1 leads to the development of cancer and cardiac hypertrophy [10–12]. The mRNA levels of both *Rrm2* and *Prkd1* were higher in A/J than in A/J-12^SM^ mice (Fig. 3a), but occurrence of liver cancer in A/J mice has not been reported. At present, the relationship between these genes and lipid metabolism remains uncertain.

Based on the results of the DNA microarray analysis, we focused on the *Iah1* gene (Table 2). A previous study reported that mouse *Iah1* mRNA was detected in the rostral striatum, kidney, liver, and lung [13]. Fukuda et al. reported that the yeast IAH1 protein has esterase activity for acetate esters such as isoamyl acetate and isobutyl acetate [6]. We found that the *Iah1* gene was widely expressed in mouse tissues (liver, kidney, epididymal fat, lung, spleen, muscle) (Fig. 3b), and the mRNA levels in the liver and kidney in A/J mice were especially low compared to those in A/J-12^SM^ mice. However, there has been no report about the function of the mouse Iah1 protein. We focused on the *Iah1* mRNA level, because the hepatic *Iah1* mRNA level was markedly low in A/J mice (Fig. 3). In this study, we constructed Hepa1-6 cells stably overexpressing the mouse Iah1 protein and found that overexpression of the Iah1 protein suppressed the expression levels of lipid metabolism-related genes such as *Cd36* and *Dgat2* (Fig. 4). Hepatic *Cd36* mRNA expression in C57BL/6 N mice was up-regulated by feeding with the HFD (82 % of calories as fat) for 2 weeks [14]. Diet-induced obesity caused an elevation of hepatic *Cd36* expression, and this elevation was correlated with the increase in liver TG storage. *In vivo*, adenoviral gene delivery of *Cd36* to the mouse liver has been shown to lead to an increase in hepatic fatty acid uptake and TG storage in the liver [15]. From our DNA microarray data, the hepatic *Cd36* mRNA level in A/J mice was about 2.6-fold higher than that in A/J-12^SM^ mice. In contrast, the mRNA levels of the other genes (*Srebf1, Gpam,* and *Dgat2*) regulated by Iah1 overexpression (Fig. 4c) were not different between these two strains (<0.667-fold or >1.5-fold). Our data implied that the elevation of the *Cd36* mRNA level in liver was correlated with the increase of hepatic TG accumulation. TG is synthesized via the acylation of diacylglycerol by DGAT enzymes (Dgat1 and Dgat2). Overexpression of the Dgat2 protein in mice has been reported to increase hepatic TG synthesis [16]. *Dgat2*-knockout mice die soon after birth because of the significant reduction in basal TG synthesis [17]. *Dgat2* antisense oligo (ASO) treatment of HFD-induced obese C57BL/6 J mice and *ob/ob* mice suppresses *Dgat2* mRNAs in the liver, resulting in a reduction in hepatic TG [18]. In addition, *Dgat2* ASO treatment in rats with diet-induced hepatic steatosis was shown to reduce hepatic diacylglycerol and TG contents, but *Dgat1* ASO treatment did not reduce either [19]. These reports indicate that the reduction of the mRNA levels of *Cd36* and *Dgat2* in liver contributes to the suppression of lipid accumulation in the liver.

We speculate that the elevation of *Iah1* gene expression suppresses the TG synthesis and the liver lipid accumulation via the reduction of *Cd36* and *Dgat2* expression. However, the regulatory mechanisms of *Cd36* and *Ggat2* gene expressions by *Iah1* are unknown. In *S. cerevisiae*, the IAH1 protein exhibits esterase activity against the ester bonds between alcohol and fatty acid [5, 6]. Therefore, we also speculate that the substrates and/or the metabolites that were catalyzed by the mouse Iah1 protein might regulate the expressions of genes related to lipid metabolism. Although the esterase properties of the mouse Iah1 protein are not yet known, we are currently trying to clarify the physiological substrates of this protein.

## Conclusions

This study demonstrated that *Fl1sa*, a QTL for fatty liver on the proximal region of chromosome 12, was the locus that was affected by feeding with the HFD, but not affected by the ND. We also showed that mouse *Iah1* is a candidate gene for *Fl1sa.* Finally, we showed for the first time that *Iah1* regulated the expressions of the *Cd36* and *Dgat2* genes, which play important roles in TG synthesis and lipid metabolism. In future experiments, to identify the gene responsible for *Fl1sa*, we will construct several congenic strains. By using these congenic mice, we can finely map the chromosomal region containing the responsible gene. Simultaneously, to determine whether the *Iah1* gene is responsible for *Fl1sa*, we will construct *Iah1*-knockout mice, which will contribute to the functional analysis of mouse Iah1. Based on our present findings, Iah1 might prove to be a novel mediator of lipid metabolism.

## Methods

### Animals

The A/J-Chr12^SM^ consomic strain was produced at the Institute for Laboratory Animal Research, Nagoya University School of Medicine. The A/J-12^SM^ consomic mouse strain was established by introduction of the donor SM/J chromosome 12 into the recipient A/J background as previously described [20]. A/J male mice were purchased from Japan SLC (Hamamatsu, Japan). In this study, only male mice were used for the phenotypic analyses. Mice were maintained in a temperature-controlled room (23 ± 2 °C) and 55 ± 5 % humidity with a 12-h light/dark cycle and ad libitum access to food and water under conventional conditions. Mice were weaned at 3 weeks of age in a cage containing five animals or fewer. Until 6 weeks of age, all mice were fed a rodent standard laboratory chow (CE2; Nihon CLEA, Japan). Animal care and all experimental procedures were approved by the Animal Experiment Committee, Graduate School of Bioagricultural Sciences, Nagoya University (approval No. 2008031801, 2011030404, 2012022805), and were conducted according to the Regulations on Animal Experiments of Nagoya University.

### Experimental schedule and diet composition

From 6 weeks of age, mice were switched from their standard laboratory diets to the HFD (HFD group), or continued to be fed their standard laboratory diets (ND group). In the ND group, A/J mice and A/J-12^SM^ mice were kept at one animal per cage and fed the standard laboratory chow for 11 weeks. After 11 weeks of feeding with the ND, the mice were killed by decapitation, and the liver was collected at 13:00–14:00 h after 4-h diet deprivation. In the HFD group, A/J mice and A/J-12^SM^ consomic mice were kept at one animal per cage and fed the powdered HFD for 3, 7, or 11 weeks. The composition of the powdered HFD (weight %) was as follows: casein, 20.9; carbohydrate (corn starch: sucrose, 1:1), 36.9; AIN93MX mineral mixture, 3.5; AIN93VX vitamin mixture, 1.0; choline chloride, 0.2; corn oil, 3.5; lard, 30.0; and cellulose (AVICEL type FD-101; Asahi Chemical Industry, Osaka, Japan), 4.0. The content of fat in this HFD was 33.5 % (weight %). After 3, 7, or 11 weeks on the HFD, mice were killed by decapitation, and the serum, liver, and fat pads were collected at 13:00–14:00 h after 4-h diet deprivation. After 7 weeks on the HFD, the measurement of BMI was performed as in our previous report [21]. BMI was calculated as body weight (g) divided by the square of the anal-nasal length (cm).

### Hepatic lipid analysis

Frozen livers were homogenized with chloroform:methanol (2:1), and the liver lipids were extracted into organic solvents. A portion of this extract was dried, and the hepatic contents of TG and TC were measured by the triglyceride E-test (Wako, Tokyo, Japan) and the cholesterol E-test (Wako), respectively. This extract was also used to measure total liver lipids according to the method of Folch et al. [22].

### Serum glucose, insulin, and lipids

The serum glucose concentration was measured by a glucose oxidase method (Glucose-B test Kit; WAKO, Tokyo, Japan). Serum insulin, TG, TC, HDL-C, and non-esterified free fatty acid concentrations were measured at the end of the experiment (after 7 weeks of feeding with the HFD) by using a Mouse Insulin ELISA kit (Morinaga Institute of Biological Sciences Inc., Japan), Triglyceride-E kit (WAKO Pure Chemical Industries, Japan), Cholesterol-E kit (WAKO Pure Chemical Industries), HDL-Cholesterol-E kit (WAKO Pure Chemical Industries), and NEFA C-kit (WAKO Pure Chemical Industries), respectively.

### Microarray experiments

Liver samples were collected from A/J male mice and A/J-12^SM^ male mice fed the HFD for 7 weeks. Total RNA was isolated from the liver using TRI reagent (Molecular Research Center Inc.) and cleaned using an RNeasy Mini kit (Qiagen). We extracted the total RNA from nine mice per strain. Extracted total RNA quality was assessed with an Agilent Bioanalyzer (Agilent Technologies). cDNA synthesis and cRNA labeling reactions were performed with a One-Cycle cDNA Synthesis Kit and IVT Labeling Kit (Affymetrix). In order to minimize the experimental variation, we used three arrays for each strain and obtained data from three replicates. Each biotinylated cRNA was prepared from the pooled RNA samples of three mice. The biotinylated cRNAs were hybridized with a probe array (Mouse Genome 430 2.0 Array) by using a Hybridization, Wash and Stain kit (Affymetrix). Scanning was performed using a Scanner 3000 with GeneChip Operating Software (GCOS) (Affymetrix). Raw data were normalized with the MAS5.0 algorithm by using Expression Console Software (Affymetrix). The microarray data have been deposited in the NCBI Gene Expression Omnibus (GEO) (GSE67340). Data were analyzed by using GeneSpring GX7.3 software (Agilent Technologies).

### Real-time RT-PCR

Total RNA was extracted from liver and Hepa1-6 cells using TRI reagent. To eliminate DNA contamination, the RNA was treated with DNase (TURBO DNA-free; Life Technologies). Subsequently, the cDNA was synthesized by using a High Capacity Reverse Transcription kit (Life Technologies). Gene expression was quantified by real-time PCR using an ABI 7300 real-time PCR System with Thunderbird qPCR Mix or Thunderbird SYBR qPCR Mix (Toyobo, Tokyo, Japan). TaqMan primers and probes were used to determine the mRNA levels of mouse Iah1 (TaqMan Gene Expression Assays, Mm00509467_m1; Applied Biosystems) and 18S rRNA (Pre-developed TaqMan Assay Reagents, Eukaryotic 18S rRNA 4319413E; Applied Biosystems). The primers used for the SYBR Green assay are shown in Additional file 1. The level of each mRNA was normalized to that of the corresponding 18S rRNA.

### Western blotting

The tissues and cells were homogenized in lysis buffer (10 mM Tris (pH 7.4), 150 mM NaCl, 1 % Nonidet P-40, 0.5 % sodium deoxycholate, 0.1 % SDS) containing protease inhibitor cocktail (Complete Mini, Roche Applied Science) with a homogenizer (HG-30; Hitachi). The homogenates were centrifuged at 10,000 × g for 5 min and the supernatant was obtained as protein extracts. An aliquot of protein (4 μg) was subjected to SDS-PAGE on 10 % acrylamide gel, and the proteins in the gel were transferred onto PVDF membranes (Hybond P; GE Healthcare). The membranes were incubated for 30 min at room temperature with Blocking One (Nacalai Tesque) and incubated overnight at 4 °C with the first antibody, rabbit polyclonal anti-mouse Iah1 (1:20,000) and anti-β-actin (1:10,000; #4967; Cell Signaling Technology Inc.), then washed with TBS buffer containing 0.1 % Tween 20. The membranes were incubated with the horseradish peroxidase-conjugated goat anti-rabbit IgG antibody (1:20,000 or 1:10,000; #7074; Santa Cruz Biotechnology, USA) for 1 h at room temperature and washed. Each antibody was diluted with Can Get Signal (Toyobo). The membranes were autographed with a West Dura Western Blot Detection kit using the ECL method (Thermo Fisher Scientific). Each protein on the band was quantified with Image J software.

### Hepa1-6 cell culture and overexpression of mouse Iah1

Hepa1-6 cells, a mouse hepatocyte cell line (RBRC-RCB1638 RIKEN BRC Cell Bank), were grown in high-glucose Dulbecco’s modified Eagle’s medium (DMEM; Wako Pure Chemical) supplemented with 10 % fetal bovine serum (FBS), 100 U/ml penicillin, and 100 μg/ml streptomycin at 37 °C in a 5 % CO_2_-humidified incubator.

Iah1 cDNA of SM/J mice was amplified with the KOD-plus (TOYOBO) using the following primers: an upper primer containing the start codon, CTTTCTACCATGTCGCTGTGC; a lower primer containing the stop codon, CTAATAGTCTCCATCTCCCAGCAG. The cDNA fragment was subcloned into pcDNA3.3 TOPO plasmid (Thermo Fisher Scientific). The Iah1-expressing plasmid vector with CMV promoter contained Iah1 cDNA (759 bp) constructed from SM/J mice. To construct the mouse Iah1-expressing stable cell line, Hepa1-6 cells were seeded on 12-well plates (3 × 10^5^ cells per well) on day 0. After 24 h, the *Iah1*-expressing plasmid vector (1.4 μg) was transfected using Lipofectamine 2000 reagent (Invitrogen). After 28 h of transfection, the transfected cells were subcultured on a 60 mm dish (6000 cells per dish) and incubated for 1 week. For the selection of neomycin-resistant cells, cells were maintained in medium containing 500 μg/ml G418 disulfate salt (Sigma Aldrich). Three weeks after colony selection with G418, we obtained the Iah1 cDNA-expressing stable cell line.

To assay the effect of overexpression of Iah1 on various mRNA levels, cells were plated on 12-well plates (2 × 10^5^ cells per well) on day 0. After 72 h, the cells were harvested and used for the assay of protein or the extraction of total RNA.

To examine the effect of overexpression of Iah1 on the intracellular accumulation of triglycerides, cells were seeded on 12-well plates (2.5 × 10^5^ cells per well) at day 0. At day 1, the culture medium was replaced with serum-free medium containing 0.333 mM oleic acid (oleic acid-BSA conjugated solution, finally containing 1 % BSA, (Sigma)) or serum-free medium alone. The treatment medium without oleic acid-BSA was supplemented with 1 % BSA. After 2 days, the cells were homogenized in lysis buffer (50 mM Tris–HCl pH 7.4, 150 mM NaCl, 2 mM EDTA, 1 % Triton X-100, and 0.5 % cholate). The supernatants were obtained by centrifugation at 9730 × g for 10 min at 4 °C. Their triglycerides and protein concentration were measured by a Triglyceride-E test and a DC protein assay (Bio-Rad Laboratories, Japan), respectively. The triglycerides content in cells was normalized to that of protein content.

### Statistical analysis

The results are expressed as the means with their standard errors. Mean values, except for the data in Fig. 4d, were compared using Student’s *t-*test when the variances of each group were equal. When the variances of each group were unequal, the significance of differences was determined using Welch’s test. The data from Fig. 4d were analyzed by two-way ANOVA. Differences with *P* < 0.05 were regarded as significant. General statistical analyses were also performed using StatView version 5.0 software (SAS Institute).

## Abbreviations

Atgl, Adipose triglyceride lipase; AUC, Area under the curve; BMI, Body mass index; Cd36, CD36 antigen; Dgat2, Acyl-CoA:diacylglycerol acyltransferase 2; FFA, Free fatty acid; Gpam, Glycerol-3-phosphate acyltransferase, mitochondrial; HFD, High-fat diet; Iah1, isoamyl acetate-hydrolyzing esterase 1 homolog (*S. cerevisiae*); Mtp1, Microsomal transfer protein 1; ND, Normal diet; Pparg2, Peroxisome proliferator-activated receptor γ; Srebf1, Sterol regulatory element binding protein 1

## Additional file

Additional file 1: The primers for SYBR Green assay in real-time PCR. (DOCX 14 kb)
