# Supplementary material for: Detection of differentially expressed candidate genes for a fatty liver QTL on mouse chromosome 12
Source: BMC Genet. 2016 Jun 6;17:73. doi: 10.1186/s12863-016-0385-2 (PMC4895971; doi:10.1186/s12863-016-0385-2)
Supplement: Additional file 1: — The primers for SYBR Green assay in real-time PCR. (DOCX 14 kb) [file 12863_2016_385_MOESM1_ESM.docx]

Additional file 1. The primers for SYBR Green assay in real-time PCR

| Gene symbol | Forward primer | Reverse primer |
| --- | --- | --- |
| *Cd36* | AATTAGTAGAACCGGGCCAC | CCAACTCCCAGGTACAATCA |
| *Dgat2* | CCTTCCTGGTGCTAGGAGTG | CCAGTCAAATGCCAGCCA |
| *Gpam* (GPAT1) | TCATCCAGTATGGCATTCTCACA | GCAAGGCCAGGACTGACATC |
| *Mttp* (MTP1) | GCTCCCTCAGCTGGTGGAT | CAGGATGGCTTCTAGCGAGTCT |
| *Pnpla2* (ATGL) | TATCCGGTGGATGAAAGAGC | CAGTTCCACCTGCTCAGACA |
| *Pparg* (PPARγ) | GCCCACCAACTTCGGAATC | TGCGAGTGGTCTTCCATCAC |
| *Srebf1* (SREBP-1c) | ACGGAGCCATGGATTGCAC | TGTCTCACCCCCAGCATAG |
